# The Effects of Yoga on Patients with Parkinson's Disease: A Meta-Analysis of Randomized Controlled Trials

**DOI:** 10.1155/2021/5582488

**Published:** 2021-07-05

**Authors:** Mengke Ban, Xuejing Yue, Pengyu Dou, Ping Zhang

**Affiliations:** ^1^Department of Neurology, The First Affiliated Hospital of Xinxiang Medical University, Xinxiang, China; ^2^Xinxiang Medical University, Xinxiang, China

## Abstract

**Methods:**

A meta-analysis was conducted by systematically searching PubMed, Embase, and Cochrane Library databases till August 2020 for studies published in English. The reference lists of eligible studies were also searched. The motor symptoms (UPDRS-Part III), balance function (BBS and BESTest), functional mobility (TUG), anxiety (HADS and BAI), depression (HADS and BDI), and the quality of life (PDQ-39 and PDQ-8) were the primary evaluation indexes.

**Results:**

Ten studies including 359 participants were included in this meta-analysis. The pooled results showed significant difference between the yoga training group and the control group. Patients in the yoga training group had better functional outcomes in terms of motor status (MD = −5.64; 95% CI, -8.57 to -2.7), balance function (SMD = 0.42; 95% CI, 0.08 to 0.77), functional mobility (MD = −1.71; 95% CI, -2.58 to -0.84), anxiety scale scores (SMD = −0.72; 95% CI, -1.01 to -0.43), depression scale scores (SMD = −0.92; 95% CI, -1.22 to -0.62), and QoL (SMD = −0.54; 95% CI, -0.97 to -0.11).

**Conclusion:**

Our pooled results showed the benefits of yoga in improving motor function, balance, functional mobility, reducing anxiety and depression, and increasing QoL in PD patients.

## 1. Introduction

PD is the second most prevalent neurodegenerative disease worldwide, only behind Alzheimer's disease [1]. It primarily occurs in middle-aged and elderly people, influencing about 1% of the population above the age of 60. The clinical manifestations associated with PD consist of motor symptoms and nonmotor symptoms. Bradykinesia, rigidity, static tremor, and postural instability are typical motor symptoms [2], which in turn affect the functional mobility, balance, and gait of patients, as well as increase the risk of falling. Anxiety, depression, and cognitive impairment are pervasive nonmotor symptoms [3], which subsequently have an immense impact on QoL.

As a chronic progressive disease, PD is mainly treated with drugs combined with routine rehabilitation training. The main purpose of the treatment is to slow down the progression of the disease, reduce the clinical symptoms, and improve QoL in PD patients. However, long-term medication may lead to several motor complications [4]. Previous studies have revealed that rehabilitation therapy, as an adjuvant strategy, could slow down the progression of PD and improve motor function, functional mobility, balance, and the health-related QoL of the patients. Holistic interventions that address both motor and nonmotor symptoms of PD simultaneously are gaining popularity. In 2002, the National Center for Complementary and Alternative Medicine of the United States conducted a survey on adults aged over 18 years, which revealed that yoga had become the fifth most frequently used method of rehabilitation therapy [5]. Yoga, a method of physical and psychological exercise and rehabilitation, has been widely used worldwide. It consists of postures (asanas), breathing (pranayama), and meditation (dhyana). Some studies have suggested that yoga can produce great benefits in ameliorating both motor and nonmotor symptoms of PD patients.

Although recent studies have reviewed the efficacy of yoga in the treatment of PD [6–8], a comprehensive view with regard to its therapeutic effects has not been reported. Some randomized clinical trials (RCTs) have evaluated the impact of yoga on motor as well as nonmotor function in PD, but the results were controversial. Ni et al. [9] and Bega and Stein [10] used Unified Parkinson's Disease Rating Scale (UPDRS), Berg Balance Scale (BBS), and Timed “Up & Go” (TUG) test to evaluate the motor functions of PD patients, and significant improvement was observed after yoga exercise. Bega and Stein [10], Kwok et al. [11], and Boulgarides et al. [12] mentioned the benefits of yoga on anxiety. However, the study conducted by Myers et al. [13] showed no positive results. To investigate whether yoga was beneficial to PD patients, and in what ways it could ameliorate the symptoms of patients and improve their quality of life, a meta-analysis of published RCTs on the effects of yoga in the treatment of PD, including motor functions, balance, mobility, anxiety, depression, and QoL, was conducted.

## 2. Methods

### 2.1. Literature Search

Two reviewers (Ping Zhang and Mengke Ban) independently searched PubMed, Embase, and Cochrane Library databases up to August 2020 with the following search terms: “Parkinson's disease” and “yoga.” The complete search strategy was described in the appendix. In addition, the list of references in all eligible articles was also reviewed to identify other relevant articles. Any disagreements during the literature search process were addressed by discussion with a third reviewer (Xuejing Yue).

### 2.2. Study Eligibility

The inclusion criteria were as follows: (1) studies designed as RCTs; (2) participants diagnosed with PD (according to the standard of MDS-UPDRS method), rather than Parkinson's syndrome; (3) participants should be divided into at least two groups, the experimental group with yoga intervention and the control group (i.e., resistance training, proprioceptive training, conventional balance exercise, waiting-list, routine care, and nonexercise control); and (4) the study should report at least one preestablished outcome. Studies were excluded if they met the following criteria: (1) case reports, conference abstracts, reviews articles, unrelated studies, comments, and cross-sectional studies; (2) duplicate articles and studies lacking relevant outcomes; and (3) studies published in non-English language.

### 2.3. Quality Assessment

To assess the quality of the included studies, the researchers independently evaluated the risk bias using Review Manager *5.4*, which involved seven domains of bias: random sequence generation (selection bias), allocation concealment (selection bias), blinding of participants and personnel (performance bias), blinding of outcome assessment (detection bias), incomplete outcome data (attrition bias), selective reporting (reporting bias), and other sources of bias (other bias). Each domain was judged as one of these three risk categories: low risk of bias, unclear risk of bias, and high risk of bias. If there was considerable controversy during the abovementioned processes, a consensus was reached by discussion with a third reviewer (Xuejing Yue).

### 2.4. Data and Variables Extraction

The following baseline data from the included literature were extracted: first author and publication year, study design, study region, characteristics of participants, experimental intervention, control intervention, and outcome measures. The outcome variables of yoga and control groups collected included the mean and standard deviation of prepost changes of UPDRS-III scores, BBS scores, TUG scores, BEST scores, HADS scores, BAI scores, BDI scores, and PDQ scores. The Unified Parkinson's Disease Rating Scale-Part III (UPDRS-Part III) was used to evaluate the motor function, which ranged from 0 to 44, with higher scores indicating worse parkinsonism. The balance was assessed by BBS and Mini-Balance Evaluation Systems Test (Mini-BESTest). The former included 14 items with a total score of 56, and the latter contained 14 motor functional tests, with the higher scores indicating higher level of balance. The TUG measured functional mobility, and the lower time indicated better mobility. The Hospital Anxiety and Depression Scale (HADS) was used to assess the symptoms of anxiety or depression, and a high score was associated with high level of psychological pain. Anxiety and depression were assessed by Beck Anxiety Inventory (BAI) and Beck Depression Inventory (BDI), with higher scores indicating the more severe symptoms. The Parkinson's Disease Questionnaire-39 (PDQ-39) and the Parkinson's Disease Questionnaire-8 (PDQ-8) were validated tools used for assessing QoL, and the lower scores indicated an improved QoL.

### 2.5. Statistical Analysis

For continuous data, the mean differences (MDs) or standardized mean differences (SMDs), with 95% confidence intervals (CIs), were calculated using RevMan *5.4.* If the mean or standard deviation of the changes was not provided, then Cochrane software system was used for the calculation of MD or SMD. Besides, chi-square test and Higgins *I*^2^ statistics were used to assess the heterogeneity across studies. An *I*^2^ value of 50% or higher was considered an indicator of substantial heterogeneity, and a random-effects model would be adopted. Meanwhile, subgroup analysis was implemented to explore the potential sources of heterogeneity. The fixed-effects model was used when the *I*^2^ value was less than 50%, indicating that the heterogeneity among the studies was not significant. All reported probability (*P*) values were two-sided, and *P* ≤ 0.05 was considered statistically significant.

## 3. Results

### 3.1. Search Results

**Figure 1:**
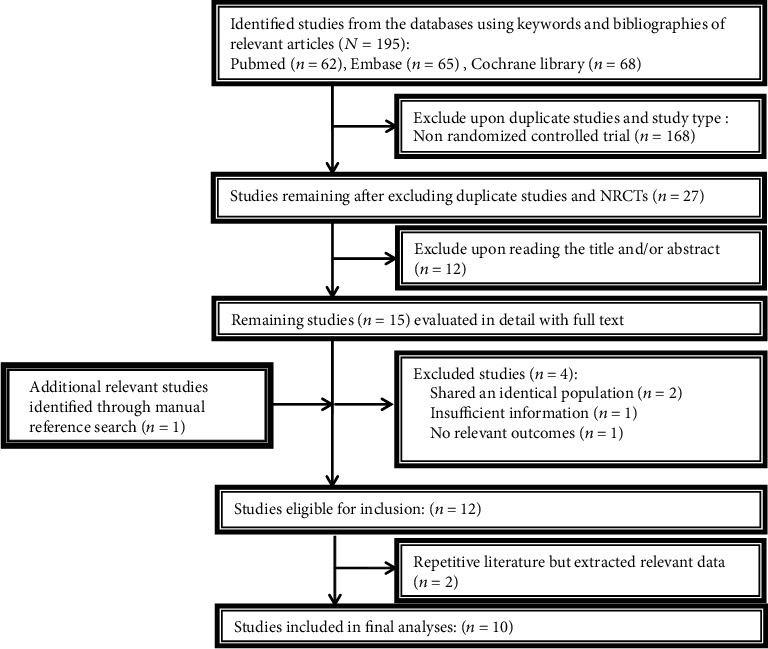
Flowchart of study selection.

Figure 1 summarized the process of literature search. A total of 62 relevant documents were identified from PubMed, Embase, and Cochrane Library databases, and 35 non-RCT articles were removed. Basing on the PICO format, the title and abstracts of these documents were carefully screened; then, 16 articles which met the eligibility criteria were selected. After reviewing the full text, 2 studies published by Sharma et al. [14] were found to come from the same trial, with each study containing different outcome measures. The article was excluded because there were no corresponding outcome measures, and the one by Colgrove in 2012 was retained. Kwok et al. [11, 15] have published 2 articles from the same trial. The publication in 2017 [15] was excluded because it was a study protocol, while the paper published in 2019 [11] was classified as eligible. Ni et al. [9, 16] reported 2 repetitive articles originated from the same trial, and both studies had their own unique outcome indicators. Our meta-analysis extracted different outcome variables from these two articles and regarded them as the same study. Hawkins et al., Van Puymbroeck et al., and Walter et al. [17–19] published 3 articles for the same trial, and a mixed study was excluded, and the outcome variables needed were extracted from the remaining 2 articles [18, 19], which were also regarded as the same study. Finally, a total of 10 studies were included.

### 3.2. Study Characteristics

A total of 359 participants were included in our meta-analysis. All subjects in the intervention and the control group were diagnosed with PD, with a Hoehn & Yahr scores ranging from 1 to 3. The average age of the patients ranged from 60 to 80 years. Furthermore, most of the studies were conducted in America. All studies were compared between the trial group (yoga exercise) and the control group (i.e., resistance training, proprioceptive training, conventional balance exercise, waiting-list, routine care, and nonexercise control). The baseline characteristics of all patients were summarized in Table 1.

### 3.3. Quality Assessment

**Figure 2:**
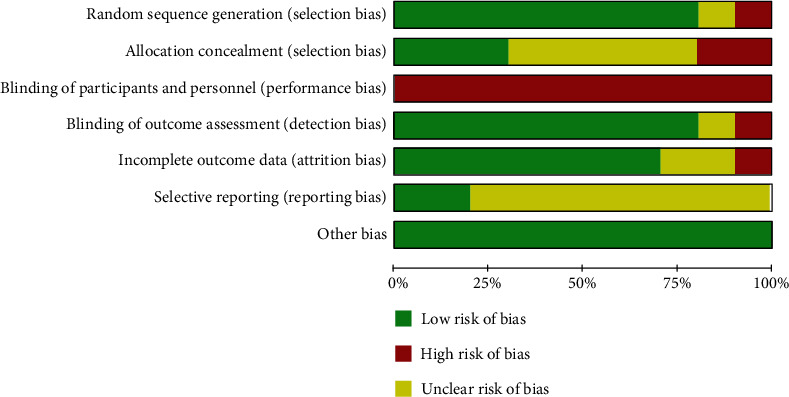
Graph of the risk of bias: percentages across all included studies.

A total of 10 RCTs evaluated the risk of bias, and 8 trials (80%) generated sufficient randomization sequence and detailed information about randomization. Allocation concealment was mentioned in 3 trials (30%). It was not possible to blind the participants to group allocation, but the system evaluator's judgment of outcome was almost unlikely to be affected by the lack of blind method. Therefore, performance bias was unavoidable in all trials. In addition, 8 trials (80%) reported the blinding of outcome assessment, and 7 trials (70%) provided complete data for the outcomes. Regarding selective reporting bias, only 2 trials (20%) met the requirements. The overall quality of included studies was medium to low (Figure 2).

### 3.4. Outcomes

#### 3.4.1. Motor Symptoms

**Figure 3:**
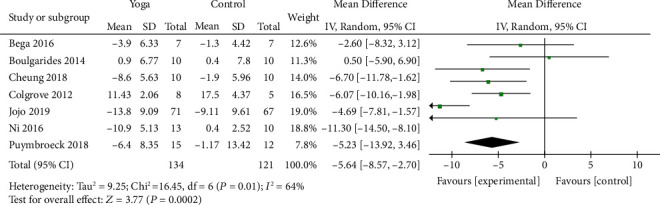
Forest plot from the meta-analysis of yoga on motor status.

**Figure 4:**
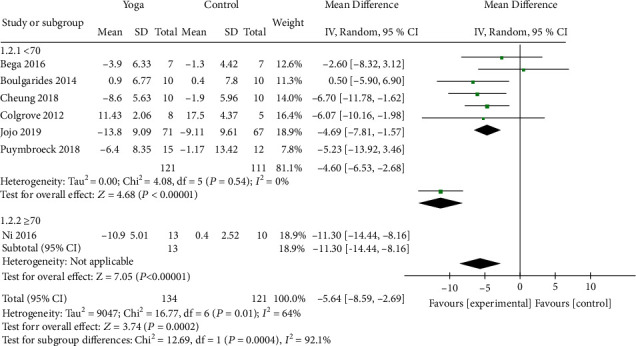
Subgroup analysis of differences among studies in UPDRS-III rating results.

Seven studies [9–12, 18, 21, 22] with 255 patients assessed motor status by UPDRS-III. A significant improvement in UPDRS-III scores was observed among the patients who performed yoga when compared to those in the control group (MD = −5.64; 95% CI, -8.57 to -2.7; *P* = 0.0002). *I*^2^ analyses revealed significant heterogeneity (*I*^2^ = 64%; *P* = 0.01), so random-effects model was used. According to sensitivity analysis, *I*^2^ was reduced to 0% when the article conducted by Ni et al. [9] was omitted (Figure 3). Subgroup analysis showed that age was the source of significant heterogeneity (Figure 4).

#### 3.4.2. Balance Function

**Figure 5:**
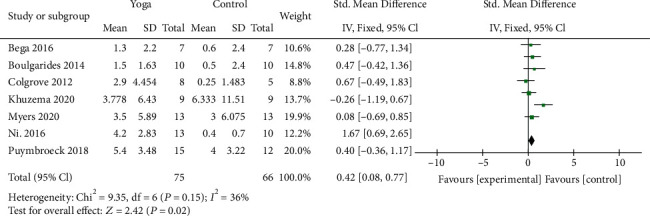
Forest plot from the meta-analysis of yoga on balance function.

Seven studies [9, 10, 12, 13, 18, 22, 23] with 141 patients evaluated the balance function of PD, among which 5 studies were assessed by BBS scores and 2 studies used the BESTest scale to evaluate the balance function. Compared with the control group, the pooled results showed that yoga intervention significantly promoted postural instability (SMD = 0.42; 95% CI, 0.08 to 0.77; *P* = 0.02). The heterogeneity test results of the included literatures showed no significance (*I*^2^ = 36%; *P* = 0.15) (Figure 5).

#### 3.4.3. Functional Mobility

**Figure 6:**
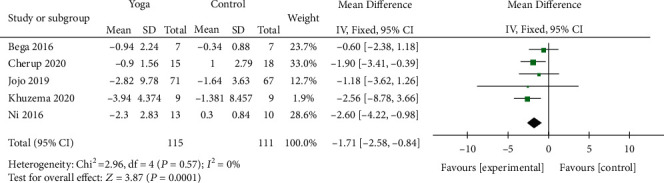
Forest plot from the meta-analysis of yoga on functional mobility.

Five studies [9–11, 20, 23] involving 226 patients assessed the functional mobility by TUG. The results (MD = −1.71; 95% CI, -2.58 to -0.84; *P* = 0.0001) demonstrated that yoga had a significant effect on improving the functional mobility of PD. There was no evidence of heterogeneity (*I*^2^ = 0%; *P* = 0.57) (Figure 6).

#### 3.4.4. Anxiety Scale Scores

**Figure 7:**
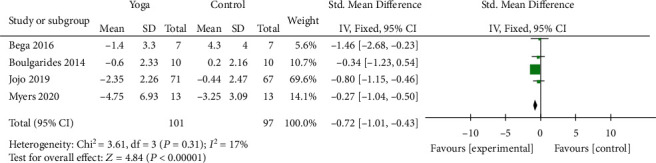
Forest plot from the meta-analysis of yoga on anxiety.

Four studies [10–13] covering 198 patients assessed nonmotor symptoms by anxiety scale scores. Among these, 2 studies used HADS and 2 studies used BAI to evaluate the efficacy of yoga on anxiety symptoms. The results showed that the scores were significantly decreased with yoga when compared to the control (SMD = −0.72; 95% CI, -1.01 to -0.43; *P* < 0.00001). There was no significant heterogeneity among the studies (*I*^2^ = 17%; *P* = 0.31) (Figure 7).

#### 3.4.5. Depression Scale Scores

**Figure 8:**
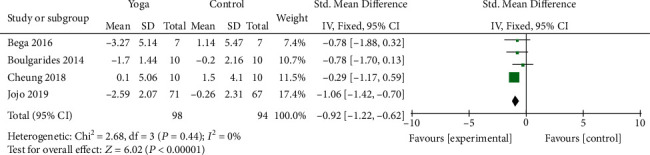
Forest plot from the meta-analysis of yoga on depression.

Four studies [10–12, 21] covering 192 patients assessed nonmotor symptoms by depression scale scores. Two studies used HADS, and two used BDI to assess the depression symptom. The pooled results suggested a significant improvement in depression for patients in the yoga exercise group compared to the control group (SMD = −0.92; 95% CI, -1.22 to -0.62 *P* < 0.00001). There was no significant heterogeneity (*I*^2^ = 0%; *P* = 0.44) (Figure 8).

#### 3.4.6. Quality of Life

**Figure 9:**
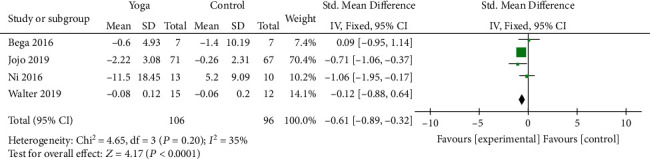
Forest plot from the meta-analysis of yoga on quality of life.

Four studies [9–11, 19] involving 202 patients evaluated QoL. Two studies used PDQ-8 to evaluate QoL, and the other 2 studies used PDQ-39 scale. The results indicated that yoga yielded significant improvement in terms of QoL of PD patients (SMD = −0.54; 95% CI, -0.97 to -0.11; *P* = 0.01). No substantial heterogeneity was detected among the studies (*I*^2^ = 35%; *P* = 0.20) (Figure 9).

## 4. Discussion

This meta-analysis was aimed at systematically evaluating the effects of yoga on patients with mild to moderate PD based on 10 eligible RCTs, including motor symptoms, nonmotor symptoms, and QoL. As we all know, exercise is an integral part of PD management, which has been shown to positively affect cognition, mood impairments, mobility, and balance and to slow the progression of PD [24, 25]. However, traditional aerobic or resistance-based exercises require safety monitoring, and some workout need exercise equipment. Yoga is generally used by the public as a way of exercise that promotes health and wellness. Through combining sitting and standing postures with breathing and meditation techniques, yoga has proven to be beneficial to health [26]. Due to its gentle approach, yoga has shown promise as an intervention that can be appropriate for PD patients who may not be able to participate in strenuous or intensive exercise [21]. Our meta-analysis suggested that yoga might have beneficial effects on promoting motor status, functional mobility, balance function, anxiety, depression, and QoL in PD patients.

Few studies have investigated the influence of yoga on motor status of PD patients, and the results of this study showed a significant improvement in UPDRS-III scores, but the heterogeneity among the articles was high. Random-effects model was adopted, and sensitivity analysis was conducted to explore the source of heterogeneity. By eliminating individual study one by one, the decrease of *I*^2^ to 0% was observed when the article conducted by Ni et al. [9] was removed, which meant that the heterogeneity between articles disappeared. Subgroup analysis showed that the average age of patients in the yoga group was over 70 years old in the study by Ni et al., while PD patients performing yoga exercise in other studies had an average age below 70 years old (Figure 4). Therefore, elderly age may be associated with the effect of yoga on motor status in PD. Studies have suggested that stretching exercises and prolonged physical postures in yoga can lengthen major muscle groups and activate the stretch receptors in muscles, ligaments, and joints, thereby improving physical strength and flexibility [27, 28]. The practice of yoga stretching posture could help to fully exercise the muscle strength of erector spinae, gluteus maximus, and rectus abdominis, increasing the stretching distance of the body, expanding the range of joint activities, and enhancing the flexibility of the lumbar, knee, and ankle joints [29]. Based on the abovementioned results, yoga can make the limb and joint activities of the individuals with PD more flexible, then reduce bradykinesia and rigidity, and increase the muscle strength and power. Moreover, yoga practice has demonstrated the greatest effect in improving motor symptoms in terms of UPDRS-III. These results were consistent with our findings.

Our meta-analysis also indicated that yoga had a significant effect on the improvement of TUG and balance function in PD. Patients with PD have an increased risk of falling due to decreased balance, reduced muscle strength, and freezing gait. The fear of falling (FOF) refers to decreased self-efficacy or low self-confidence to avoid falling during activities [30]. Studies have revealed that yoga could significantly improve the sensitivity of muscle proprioception, the stability of vestibular system, and the comprehensive analysis ability of cerebral cortex of PD patients by altering the fast and slow rhythms [31], so yoga intervention was shown to be successful in reducing the levels of FOF in individuals with PD. Nevertheless, yoga was considered as an alternative therapy, and there was evidence that balance training in yoga could make patients with PD more stable, straighter, and better at switching and anticipating movements. Many yoga postures challenge balance by placing the body in an unstable position, which in turn cannot be maintained without appropriate muscular activation to stabilize the joints [13].

Anxiety is a common nonmotor symptom that can affect up to 55% of patients with PD [32]. Previous studies have mentioned the benefits of yoga on anxiety, whereas the study by Myers et al. [13] showed that there was no significant decrease in BAI in the yoga group. Therefore, a more detailed statistical analysis was conducted, and the pooled results demonstrated that yoga could significantly ameliorate the symptoms of anxiety in patients with PD. Some studies have found that depression has a negative impact on energy levels, concentrating and decision-making, sleep, and perceived quality of life. Moreover, motor and cognitive competence were also negatively affected by depression in those with PD [33, 34]. A statistically significant effect on depressive symptoms in patients with PD was observed in our meta-analysis. There was evidence that yoga exercise could correct underactive parasympathetic system and GABAergic system through vagal nerve stimulation, thereby reducing the allostatic load in the stress response system, which in turn might affect the brain's interpretation and response to internal stress and might enhance stress resilience [35, 36]. Yoga has also been shown to decrease cortisol level and increase GABAergic activity, thereby improving patient mood and reducing anxiety [37, 38]. The reduction of anxiety and depressive symptoms in the yoga group showed significant differences. However, due to the limitations in the number of articles and sample size, further studies were warranted to strengthen and consolidate these evidences.

Some evidences showed that the QoL of patients with PD was affected by functional impairment and nonmotor symptoms, which had a serious negative consequence for patients with PD. Our meta-analysis suggested that yoga significantly improved QoL of PD patients. In short, yoga could improve motor symptoms, reduce the emotions of anxiety and depression, increase QoL, and promote the development of physical and mental health of PD patients.

One of the included articles [11] reported adverse events. Both the yoga group and the control group demonstrated transient mild knee pain, but they required no medical attention. None of the included articles reported serious adverse events.

However, our study had some limitations, which were as follows: firstly, the loss of research protocol and trial registration information in most of the articles could introduce reporting bias. Publication bias might exist because funnel plots could not be evaluated due to inclusion of small number of studies. Secondly, due to the inclusion of fewer studies, the sample size of our meta-analysis remained small, and some meaningful outcome measures were not analyzed in this study, such as Giladi Freezing of Gait Scale (FOG), 10-Minute Walk test, and Montreal Cognitive Assessment (MoCA). Future studies should delve into these aspects to find out more benefits of yoga in the treatment of PD. Thirdly, most of the studies we included did not compare yoga with other complementary approaches such as Tai Chi, Tango, and Qi Gong. Whether yoga had a greater advantage than other exercises, whether it needed to be combined with other exercises, and what is the optimal amount of yoga exercise, all these needed further study. Next, we plan to make a profound study on the differences among yoga, Tai Chi, Tango, Qi Gong, and other traditional treatments. These limitations might affect the reliability of our results; therefore, our results should be interpreted with caution.

## 5. Conclusion

Our pooled results indicated that yoga might significantly improve the motor status, balance, functional mobility, and quality of life of individuals with PD and might reduce their anxiety and depression. Due to several limitations in this meta-analysis, well-designed studies with larger sample size and additional detailed outcomes were necessary to obtain clear conclusions of yoga on PD.

## Figures and Tables

**Figure 10:**
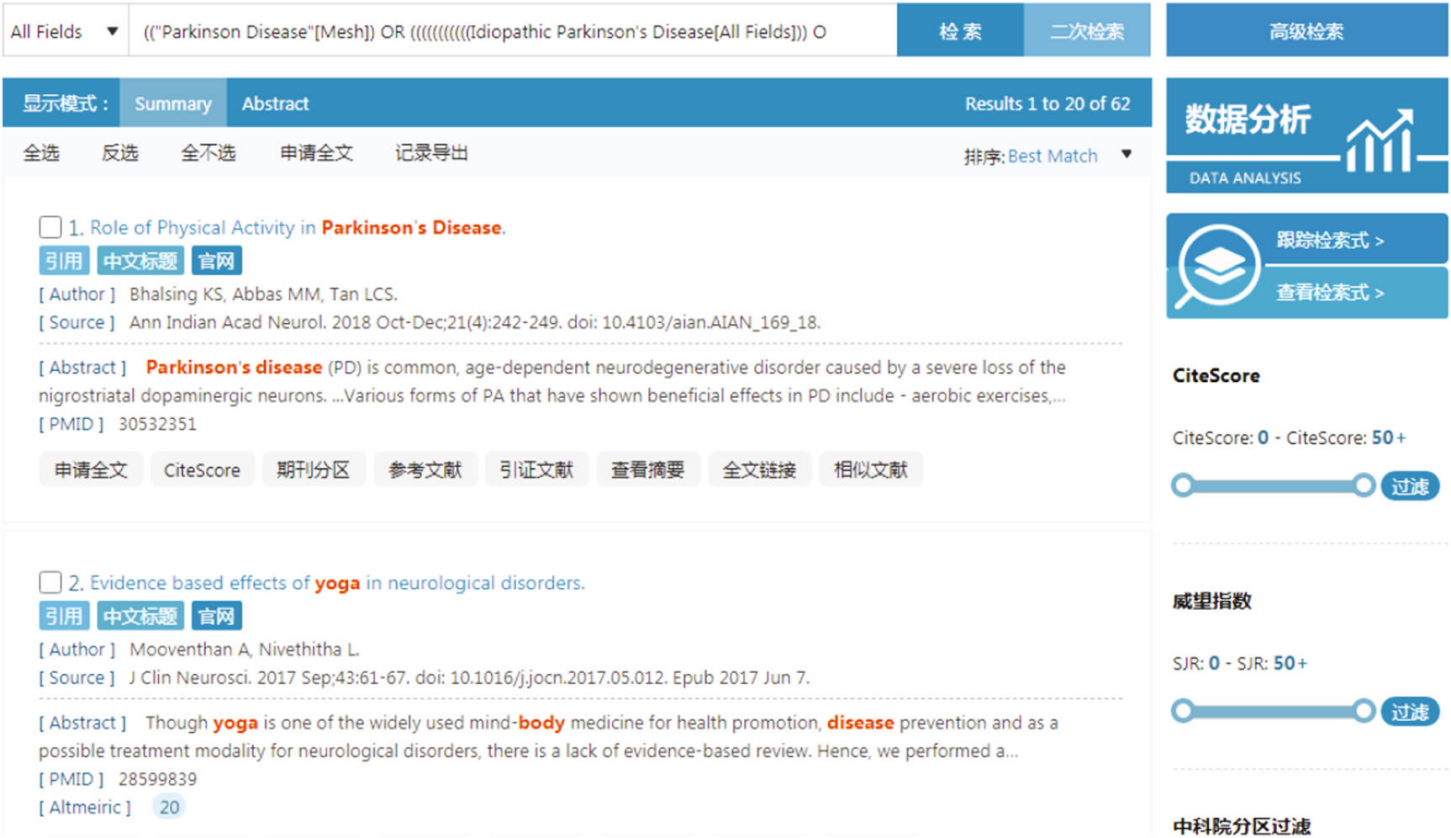
Results of literature search in PubMed database.

**Table 1:** Characteristics of included studies.

Study	Region	Study design	Participants and group allocation	Intervention	Control	Main outcome measures
Bega et al. (2016) [10]	USA	RCT	Experimental group (*n*: 7;): age (y): 67.9 ± 10.9; gender (M/F): 5/2; HY: 2.3 ± 0.4 Control group (*n*: 7): age (y): 66.7 ± 9.3; gender (M/F): 6/1; HY: 2.4 ± 0.5	Experimental group (yoga): yoga exercise 60 min, 2/wk × 12 wk (deep breathing exercises and relaxation techniques; pose targeting; meditation)	Control group: resistance training	TUG, UPDRS, BBS, PDQ-39, BDI
Boulgarides et al. (2014) [12]	USA	RCT	Experimental group (*n*: 10): age (y): 65.7; gender (M/F): 7/3; HY: 2.6 Control group (PD): age (y): 65.7; gender (M/F): 7/3; HY: 2.6	Experimental group (yoga): yoga exercise 60 min, 1/wk × 8 wk (postural training; meditation; deep relaxation)	Control group: wait-list	UPDRS, HADS, BBS
Cherup et al. (2020) [20]	USA	RCT	Experimental group (*n*: 15): age (y): 69.8 ± 7.3; gender (M/F): 10/5 Control group (*n*: 18): age (y): 71.4 ± 12.1; gender (M/F): 11/7	Experimental group (yoga): yoga exercise 45 min, 2/wk × 12 wk (postural training; meditation)	Control group: proprioceptive training 45 min, 2/wk × 12 wk	TUG
Cheung et al. (2018) [21]	Australia	RCT	Experimental group (*n*: 10): age (y): 63.5 ± 8.5; gender (M/F): 7/6; HY: 2 ± 0.8 Control group (*n*: 10): age (y): 65.8 ± 6.6; gender (M/F): 8/5; HY: 2 ± 0.8	Experimental group (yoga): yoga exercise 60 min, 2/wk × 12 wk (poses; breathing techniques; meditation)	Control group: the wait-list group: usual care	UPDRS, MoCA, Beck Depression Inventory
Colgrove et al. (2012) [22]	USA	RCT	Experimental group (*n*: 8): age (y): 62.8 ± 13.2; gender (M/F): 2/6; HY: 1.3 ± 0.3; duration of disease (y): 3.8 ± 2.9 Control group (*n*: 5): age (y): 73.4 ± 6.5; gender (M/F): 4/1; HY: 1.2 ± 0.4; duration of disease (y): 3.7 ± 22.2	Experimental group (yoga): yoga exercise 60 min, 2/wk × 12 wk (poses; breathing techniques; meditation)	Control group: yoga sessions 12 wk	UPDRS-III, BBS, ROM, muscle strength gait (postural sway and gait initiation)
Jojo et al. (2019) [11]	China (Hong Kong)	RCT	Experimental group (*n*: 71): age (y): 63.7 ± 8.2; gender (M/F): 37/34 Control group (*n*: 67): age (y): 63.5 ± 9.3; gender (M/F): 28/39	Experimental group (yoga): yoga exercise 90 min/wk × 8 wk (breathing exercise (15 min); mindfulness practice (15 min); yoga practice (60 min))	Control group: stretching and resistance exercises 60 min/wk × 8 wk	HADS, UPDRS-III, TUG, HWS, PDQ-8
Khuzema et al. (2020) [23]	India	RCT	Experimental group (*n*: 9): age (y): 68.11 ± 4.23; gender (M/F): 6/3; duration of disease (y): 6.2 ± 1.67 Control group (*n*: 9): age (y): 70.89 ± 6.01; gender (M/F): 7/2; duration of disease (y): 5.23 ± 3.12	Experimental group (yoga): yoga exercise 30–40 min, 8 wk (postural training; deep breathing exercises)	Control group: conventional balance exercise 30–40 min, 8 wk	BBS, TUG, 10-Minute Walk test
Myers et al. (2020) [13]	USA	RCT	Experimental group (*n*: 13): age (y): 70.5 ± 8.7; gender (M/F): 7/6; HY: 2 Control group (*n*: 13): age (y): 65.0 ± 8.7; gender (M/F): 8/5; HY: 2	Experimental group (yoga): yoga exercise 60 min, 2/wk × 12 wk (relaxation and meditation 5 minutes; gentle spinal movements 10 minutes; standing poses 30–35 minutes; cool down 5–10 minutes; rest and relaxation 5 minutes)	Control group: usual daily routines 12 wk	BESTest, BAI
Ni et al. (2016) (same study with Ni et al. 2016) [9, 16]	USA	RCT	Experimental group (*n*: 13): age (y): 71.2 ± 6.5; gender (M/F): 11/2; HY: 2.2 ± 0.7; duration of disease (y): 6.9 ± 6.3 Control group (*n*: 10): age (y): 74.9 ± 8.3; gender (M/F): 4/6; HY: 2.1 ± 0.7; duration of disease (y): 5.9 ± 6.2	Experimental group (yoga): yoga exercise 1 h, 2/wk × 12 wk (poses and deep breathing exercises)	Control group: 1-hour nonexercise, health education class,1/mo × 3 mo	UPDRS-III, BBS, Mini-BESTest, TUG, PDQ-39
Puymbroeck et al. (2018) (same study with Walter et al. 2019) [18, 19]	USA	RCT	Experimental group (*n*: 15): age (y): 65.53 ± 6.09; gender (M/F): 10/5 Control group (*n*: 12): age (y): 70.5 ± 4.44; gender (M/F): 7/5	Experimental group (yoga): yoga exercise 60 min, 2/wk × 8 wk (posture; pranayama; dhyana)	Control group: usual care 8 wk	UPDRS, Mini-BESTest FGA, FOG, PFS-16, PDQ-8

RCT: randomized controlled trial; M: male; F: female; values are mean ± SD; HY: Hoehn and Yahr scale; min: minute; wk: week; mo: month; TUG: Timed Up and Go test; UPDRS: Unified Parkinson's Disease Rating Scale; UPDRS-III: the motor subscale of the Unified Parkinson's Disease Rating Scale; BBS: Berg Balance Scale; PDQ-39: Parkinson's Disease Questionnaire-39; BDI: Beck Depression Inventory; HADS: Hospital Anxiety and Depression Scale; MoCA: Montreal Cognitive Assessment; ROM: range-of-motion; HWS: Holistic Well-being Scale; PDQ-8: Parkinson's Disease Questionnaire-8; Mini-BESTest: Mini-Balance Evaluation Systems Test; BAI: Beck Anxiety Inventory; FGA: Functional Gait Assessment; FoG: Freezing of Gait Questionnaire; PFS-16: Parkinson's Fatigue Scale-16.

## Appendix

### Search strategy

#1 (Parkinson Disease) OR (Idiopathic Parkinson's Disease) OR (Lewy Body Parkinson's Disease) OR (Parkinson's Disease, Idiopathic) OR (Parkinson's Disease, Lewy Body) OR (Parkinson Disease, Idiopathic) OR (Parkinson's Disease) OR (Idiopathic Parkinson Disease) OR (Lewy Body Parkinson Disease) OR (Primary Parkinsonism) OR (Parkinsonism, Primary) OR (Paralysis Agitans) (Figure 10)

#2 (Yoga)

#3 #1 AND #2

#4 (randomized controlled trial) OR (controlled clinical trial) OR (randomized) OR (placebo) OR (drug therapy) OR (randomly) OR (trial) OR (groups) NOT (animals) NOT (humans)

#5 #3 AND #4

Pubmed:

(((Parkinson Disease) OR (((((((((((Idiopathic Parkinson's Disease[All Fields])) OR (Lewy Body Parkinson's Disease)) OR (Parkinson's Disease, Idiopathic)) OR (Parkinson's Disease, Lewy Body)) OR (Parkinson Disease, Idiopathic)) OR (Parkinson's Disease)) OR (Idiopathic Parkinson Disease)) OR (Lewy Body Parkinson Disease)) OR (Primary Parkinsonism)) OR (Parkinsonism, Primary)) OR (Paralysis Agitans)) AND (Yoga) AND (((((((((randomized controlled trial)) OR (controlled clinical trial)) OR (randomized)) OR (placebo)) OR (drug therapy)) OR (randomly)) OR (trial)) OR (groups)) NOT ((animals)) NOT (humans).
